# Supplementary material for: Individual and community-level factors of treatment-seeking behaviour among caregivers with febrile children in Ethiopia: A multilevel analysis
Source: PLoS One. 2022 Mar 17;17(3):e0264707. doi: 10.1371/journal.pone.0264707 (PMC8929549; doi:10.1371/journal.pone.0264707)
Supplement: Attachment — Submitted filename: Response to Reviewers.docx [file pone.0264707.s005.docx]

**Authors’ Response for Reviewers’ Comments**

**Manuscript ID:** PONE-D-21-03591

**Title: Individual and community-level factors associated with treatment-seeking behavior among caregivers with febrile children in Ethiopia: A Multilevel analysis**

**Dear editor(s) and reviewers**

First for all we would like to thank you for helping us by reviewing our paper entitled, **“Individual and community-level factors associated with treatment-seeking behavior among caregivers with febrile children in Ethiopia: A Multilevel analysis”** by giving your precious time. Thus, your comments and questions are constructive and interesting for us. Based on your questions and suggestions we have already modified accordingly.

Reviewers question and/ comments

Response=> Authors response based on the reviewer(s) questions and comments

**Reviewer #1**

Thank you very much for your review. We appreciate the positive feedback from the reviewer

**Reviewer #2**

Q1: The authors have addressed many of the reviewer comments and the revised manuscript is greatly improved. The introduction does a better job at setting up the paper, the methods section has some additional information, and the results are more clearly presented and contextualized in the discussion. However, there are still some concerns with the paper and several comments from the prior round of review that were not addressed. Comments below are organized by section.

*Response*: Thank you very much for your comments and suggestions. Our revised responses based on questions and comments which may help to clarify the paper and its findings are as follows:

Methods:
1. Several of the prior comments about variables and measures were not addressed. A new section was added and some additional information was provided, but the questions/comments about variable construction, variables used in analysis not mentioned anywhere else until showing in tables, construction of indices etc. were not addressed. Each of the subitems mentioned below was brought up in the prior comments.

*Response*: we had been addressed the comments.

a. Additional information about the index variables is needed in the manuscript (see prior comments). Did the community development index and the household wealth index use the same WASH measures? Were all items weighted equally? How exactly were the indices constructed? How were cutoffs and quintiles decided upon? Are there references for these indices? Community level cluster variable based on the WASH and electricity (have other studies used this?)

*Response*: *The wealth index was computed by principal component analysis based on asset based data for urban and rural areas. While the community development index was a composite variable formed from the availability of three basic services in the community: improved water supply, electricity city, and improved sanitation services. It is classified as: Low: community with none of those three services; Medium: community with one or two services; High: community with three services.*

b. Some issues remain with inconsistencies between what variables are listed in different sections of the paper. For example, the first sentence on independent variables says “size of child at birth” is the measure, but then later in the same paragraph, the authors list under five height for age Z score, weight for age Z score and weight for height Z-score as the measures.

*Response*: *size of child at birth is to indicate birth weight whereas under five heights for age Z score, weight for age Z score and weight for height Z-score were used as index for assessing the nutritional status during life after birth.*

c. No information is given on several measures or why they may be important to include (some of this could go in intro if more appropriate) – examples include but are not limited to mass media exposure, vaccination, place of residence (is this the same as region? ), health insurance (is this yes/no? are there different types of insurance? Do you caregivers need insurance for sick child visits?), How is distance measured (km?, time to get there, ? Does it take main modes of transportation for the study population into account) – Some information can be ascertained from the tables, but the importance of these measures to the study and why they are included (and operationalized in certain ways) is missing for many of them.

*Response*: the health insurance variable is yes/no type variable and it is community health insurance. In addition, the caregivers might need health insurance because some of the services like lab investigation and medication might not fully available to the public facility. In this case the might utilize the service in private and secured their budget from their insurance. This study utilizes the DHS data from Ethiopia which measures the distance to health facility and categorized as big problem and not a big problem.

d. The authors have not adequately addressed what the “community” level is. Judging by line 187 and line 228, the community is defined as the EDHS EAs, but it would be very helpful to state this explicitly and give the number of clusters etc. in the methods section (beyond the data section that describes how the sampling was done). Also, why is health insurance a community level variable?

*Response*: community in our case is to mean a group of individuals that shared similar characteristics. As you said the community in EDHS data is EAs and also we had mentioned it on the manuscript its total size and the urban and rural EAs which was 645, 202, and 443 EAs respectively. We used the health insurance variable as community level because the type of health insurance that were collected by the EDHS data was community based health insurance.A community was defined as a group of households sharing a common primary sampling unit/cluster within the dataset. Community level were constructed by aggregating them from individual-level factors.

e. This is not a major issue – but it is indicative of overlooking specific items brought up by the reviewers: In the author response to a reviewer comment about using a cutoff of p<0.26, they said this was a typo and that it was fixed – line 181 still lists the same cutoff with the typo.

*Response*: sorry for reaped typological error but we have edited on the revised document.

f. The authors responded to a comment about missing data in the response to reviewers, but didn’t mention how they handled missing data in the manuscript – complete case analysis (with % missing)

*Response*: as we said in the previous comment, the missing data’s were handled by complete case analysis after checking the% of missing because the variables with missed data had missing value < 5%.

2. Methods: Because it is such a critical part of the study, I would suggest having a clearer sentence stating your outcome variable – example: “ The outcome variable for this study was treatment-seeking behavior (Yes/ No), defined as whether or not a caregiver sought advice or treatment from a health facility for a living child under-five who had a fever at any time in the 2 weeks preceding the survey.”

*Response*: thank you for your constructive comment, we accept it and change by your ideas

Results
1. Why not show your main outcome variable in Table 1?

*Response*: We thank the reviewer for the suggestions. We have included the suggested changes and track changed in the revised manuscript.

2. I might also suggest adding a bivariate table. It might be helpful to see some of the bivariate associations/percentages and numbers of observations. You could truncate Table 1 and also not show every variable or every level of every variable in Table 2 if space was a concern.

*Response*: We appreciate the positive feedback from the reviewer, based on your comment we have modified this section in understandable and precise way.

Editing/Grammar/Formatting:
1. The writing/grammar is much better and really improves readability, however, the manuscript still needs some copyediting. A few notes on this are below.

2. There are some typos and inconsistencies between the different versions of the paper (clean revised manuscript and the tracked changes version). I note some specific inconsistencies below, but this should be thoroughly reviewed. Having two different versions of the revised manuscript made it very difficult to re-review this paper. Which one is the most updated/accurate?

*Response*: After reviewer(s) suggestions, we have revised again the document and we also have found grammatical, contextual unclarity and sentence structure problems. Then we have consumed more time and energy for re-edition of the vague sentences. Based on this, we have rewritten the whole document in more understandable to resolve language problems.

Introduction: in the clean version, the second sentence of the Intro (lines 55-57) includes a line from the prior sentence (major typo). This seems to be ok in the track changes version. In the clean version there is a sentence on lines 73-73 “In Ethiopia healthcare-seeking behavior is poor….” This sentence does not appear in the tracked changes version

*Response*: Thank you for your comments, the suggested correction has been made.
o Paragraph delineation: Examples - in the tracked changes version, on the first page of the introduction there is a new paragraph starting with “World Health Organization.” In the clean version this is lumped in with the prior paragraph (line 68). In the clean version line 81, there is a new sentence starting with “Due to the inadequacy…” but this is an entirely new paragraph in the tracked changes version (also missing a period at the end of the prior sentence).

*Response*: It has been rewritten again by incorporating necessary information in this regard.

3. There are different fonts, spacing, sizing used throughout

*Response*: The font size and type has been checked and it is consistent in the whole document. The used font type and size is “Times New Roman” by “12”.

4. Some capitalization typos – examples include “Children” on lines 134 and 135 (additional typos/grammar issues in the line 134 sentence), Benishangul (line149)

*Response*: the correction has been made.
5. The tables need significant formatting work to be more readable – Table 1 is 4.5 pages long (unnecessarily). A reviewer previously suggested adding totals (this does not need to be for every variable – could be overall). Table 2 – one suggestion, you can delete all the lines for the

*Response*: the correction has been made.

Finally, we thank the reviewers’ editors for their kind comments, constructive criticisms and useful suggestions for which we have used to improve our manuscript. We have re-read, edit and rewrite the whole manuscript once again and made any necessary editorial corrections. Thank you for the helpful review once again.
